# Supplementary material for: Analysis of the Holarctic Dictyoptera aurora Complex (Coleoptera, Lycidae) Reveals Hidden Diversity and Geographic Structure in Müllerian Mimicry Ring
Source: Insects. 2022 Sep 7;13(9):817. doi: 10.3390/insects13090817 (PMC9502218; doi:10.3390/insects13090817)
Supplement: Supplementary file 1 [file insects-13-00817-s001.zip › insects-1892575-supplementary.pdf]

Table S1. Genetic distances among *D. aurora* barcodes

	Barcode	1	2	3	4	5	6	7	8	9	10	11	12	13	14	15	16	17	18	19	20	21	22	23	24	25	26	27	28	29	30	31	32	33	34	35	36	37	38	39	40	41	42	43	44	45	46	47	48	49	50	51	52	53	54	55	56	57	58	59	60	61	62	63	64	65	66	67
1	ASALC410-13 Canada Ontario	-																																																																	
2	BBCCN733-10 Canada Ontario HQ928222	96.5	-																																																																
3	CNLMC030-14 Canada Quebec KR119387	96.8	99.4	-																																																														
4	CNLMD1590-14 Canada Quebec KR130675	97.0	99.5	99.8	-																																																												
5	OPPAK060-17 Canada Ontario	97.0	99.5	99.8	100.0	-																																																										
6	CNLMC029-14 Canada Quebec KR128162	96.8	98.5	98.5	98.6	98.6	-																																																									
7	CNBRB543-14 Canada Nova Scotia KR126473	96.8	96.2	96.5	96.6	96.6	97.1	-																																																						
8	CNFNP180-14 Canada Quebec KR131217	97.0	96.3	96.6	96.8	96.8	97.1	99.5	-																																																					
9	CNKOM073-14 Canada New Brunswick KR127450	97.4	96.5	96.8	97.0	97.0	97.3	99.4	99.5	-																																																			
10	GMFAI077-15 United States Virginia	97.3	96.4	96.9	96.9	96.9	97.3	99.5	99.7	99.8	-																																																	
11	GMFRB450-14 United States Virginia	97.5	96.5	96.8	97.0	97.0	97.5	99.0	98.9	99.0	99.5	-																																																
12	CNLMC031-14 Canada Quebec KR121647	97.0	96.3	96.6	96.8	96.8	97.1	99.4	99.5	99.4	99.5	98.7	-																																														
13	TTCFW420-08 Canada Ontario KM843811	97.0	96.3	96.6	96.8	96.8	97.1	99.4	99.5	99.4	99.5	98.7	100.0	-																																												
14	DIMC055-09 Canada Ontario GU013593	97.0	96.3	96.6	96.8	96.8	97.1	99.4	99.5	99.4	99.5	98.7	100.0	100.0	-																																										
15	CNWLL371-13 Canada Alberta KM847614	97.4	97.1	97.4	97.6	97.6	97.7	98.5	98.3	98.8	98.5	98.7	98.2	98.2	98.1	-																																								
16	UAMIC155-13 United States Alaska KU874829	97.4	97.1	97.4	97.6	97.6	97.7	98.5	98.3	98.8	98.5	98.7	98.2	98.2	98.1	100.0	-																																							
17	UAMIC285-13 United States Alaska KU874830	96.8	96.6	96.8	97.1	97.1	97.5	98.3	98.0	98.3	97.9	98.4	98.0	98.0	97.9	100.0	100.0	-																																						
18	TTCFW261-08 Canada Ontario KM847232	97.0	97.0	97.3	97.4	97.4	97.4	98.0	98.2	98.3	98.3	98.3	98.2	98.2	98.1	98.9	98.9	99.0	-																																					
19	TTCFW421-08 Canada Ontario KM844857	97.3	97.3	97.6	97.7	97.7	97.9	98.6	98.5	98.6	98.6	98.9	98.5	98.5	98.4	99.5	99.5	99.5	99.4	-																																				
20	GMFRB451-14 United States Virginia	97.3	96.7	97.3	97.1	97.1	97.1	97.9	98.1	98.6	98.5	98.3	97.9	97.9	97.9	98.9	98.9	98.4	98.4	98.7	-																																			
21	CNGMC001-13 Canada Newfoundland and Labrador KR123415	96.6	96.1	96.2	96.2	96.2	96.9	97.9	98.0	98.2	98.3	98.4	98.2	98.2	98.2	97.5	97.5	97.2	97.4	97.7	97.5	-																																		
22	CNGMC002-13 Canada Newfoundland and Labrador KR123533	96.7	96.2	96.4	96.4	96.4	97.0	98.2	98.4	98.5	98.6	98.7	98.2	98.2	98.2	97.9	97.9	97.5	97.7	98.0	97.9	99.7	-																																	
23	CNGMC003-13 Canada Newfoundland and Labrador KR130333	96.7	95.9	96.4	96.4	96.4	96.7	98.2	98.3	98.5	98.6	98.7	98.3	98.3	98.3	97.8	97.8	97.5	98.0	98.3	97.8	99.3	99.7	-																																
24	CNSLB261-12 Canada Ontario KM845594	96.3	96.0	96.0	96.2	96.2	96.8	98.3	98.2	98.3	98.3	98.3	98.0	98.0	97.9	97.7	97.7	97.3	97.6	97.9	97.5	99.2	99.5	99.2	-																															
25	CNSLB264-12 Canada Ontario KM843181	96.3	96.0	96.0	96.2	96.2	96.8	98.3	98.2	98.3	98.3	98.3	98.0	98.0	97.9	97.7	97.7	97.3	97.6	97.9	97.5	99.2	99.5	99.2	100.0	-																														
26	SDP864010-19 United States Maine MN556953	96.5	96.2	96.2	96.3	96.3	97.0	98.5	98.3	98.5	98.5	98.4	98.2	98.2	98.1	97.9	97.9	97.5	97.7	98.0	97.6	99.3	99.7	99.3	99.8	99.8	-																													
27	CNFDC064-14 Canada New Brunswick KR123858	96.2	97.1	96.8	97.0	97.0	97.0	97.0	97.1	97.3	97.1	97.1	97.1	97.1	97.0	97.9	97.9	97.8	98.0	98.3	97.6	96.2	96.6	96.9	96.5	96.5	96.6	-																												
28	CNYOA150-15 Canada British Columbia KR915224	96.5	96.1	96.7	96.7	96.7	96.9	97.4	97.2	97.8	97.6	98.1	97.0	97.0	97.0	99.2	99.2	100.0	97.9	98.7	98.3	96.5	96.9	96.9	96.5	96.5	96.7	96.9	-																											
29	OPPUC028-17 Canada Ontario	95.0	94.4	94.8	94.8	94.8	94.5	94.5	94.5	95.0	94.9	94.9	94.7	94.7	94.7	94.8	94.8	94.6	94.5	94.8	94.9	94.4	94.6	94.9	94.5	94.5	94.4	94.2	94.2	-																										
30	COLFB289-12 Finland Finland Proper KJ964562	89.3	88.7	89.0	89.2	89.2	89.3	89.8	89.9	89.5	89.6	90.0	89.6	89.6	89.5	89.6	89.6	87.7	89.3	89.8	89.2	88.9	89.0	89.1	88.9	88.9	89.0	89.5	88.8	88.1	-																									
31	LEFIJ2370-14 Finland MZ610715	89.3	88.7	89.0	89.2	89.2	89.3	89.8	89.9	89.5	89.6	90.0	89.6	89.6	89.5	89.6	89.6	87.7	89.3	89.8	89.2	88.9	89.0	89.1	88.9	88.9	89.0	89.5	88.8	88.1	100.0	-																								
32	COLFC799-12 Finland Kainuu KJ966088	89.2	88.6	89.2	89.0	89.0	89.2	89.6	89.8	89.3	89.6	89.8	89.5	89.5	89.3	89.5	89.5	87.5	89.2	89.6	89.4	88.9	89.0	89.1	88.7	88.7	88.9	89.3	88.8	88.1	99.8	99.8	-																							
33	COLFH1047-15 Finland MZ656640	89.3	88.6	88.9	89.0	89.0	89.2	89.6	89.8	89.3	89.4	89.8	89.5	89.5	89.3	89.5	89.5	87.5	89.2	89.6	89.0	88.7	88.8	88.9	88.7	88.7	88.9	89.3	88.6	88.0	99.8	99.8	99.7	-																						
34	LEFIJ2366-14 Finland MZ607403	89.2	88.6	88.9	89.1	89.1	89.2	89.7	89.8	89.4	89.5	89.9	89.5	89.5	89.3	89.5	89.5	87.8	89.2	89.7	89.1	88.7	88.9	88.9	88.8	88.8	88.9	89.4	88.7	88.0	99.7	99.7	99.6	99.6	-																					
35	NOCOL067-12 Norway Buskerud	89.2	88.6	88.9	89.0	89.0	89.2	89.6	89.8	89.3	89.4	89.8	89.5	89.5	89.3	89.5	89.5	87.7	89.2	89.6	89.0	88.7	88.8	88.9	88.7	88.7	88.9	89.3	88.6	88.1	99.8	99.8	99.7	99.7	99.6	-																				
36	COLFB382-12 Finland Northern Ostrobothnia KJ963627	89.1	88.4	88.8	88.9	88.9	89.1	89.6	89.7	89.2	89.3	89.8	89.4	89.4	89.2	89.4	89.4	87.5	89.1	89.6	89.1	88.6	88.7	88.8	88.6	88.6	88.8	89.2	88.6	88.0	99.8	99.8	99.7	99.7	99.6	99.7	-																			
37	COLFH874-15 Finland MZ656967	89.3	88.7	89.0	89.2	89.2	89.3	89.8	89.9	89.5	89.6	90.0	89.6	89.6	89.5	89.6	89.6	88.0	89.3	89.8	89.2	88.9	89.0	89.1	88.9	88.9	89.0	89.5	89.0	88.1	99.7	99.7	99.5	99.5	99.4	99.5	99.5	-																		
38	COLFH298-14 Finland MZ610373	89.3	88.7	89.0	89.1	89.1	89.3	89.7	89.9	89.4	89.6	90.0	89.6	89.6	89.5	89.6	89.6	87.6	89.3	89.7	89.2	88.9	89.0	89.1	88.8	88.8	89.0	89.4	88.8	88.1	99.8	99.8	99.7	99.7	99.6	99.7	99.7	99.5	-																	
39	COLFG526-14 Finland MZ609875	89.5	88.9	89.5	89.3	89.3	89.5	89.9	90.1	89.6	89.9	90.2	89.8	89.8	89.6	89.8	89.8	88.0	89.5	89.9	89.7	89.2	89.3	89.4	89.0	89.0	89.2	89.6	89.2	88.4	99.2	99.2	99.4	99.1	99.3	99.1	99.1	98.9	99.1	-																
40	COLFG538-14 Finland MZ608375	89.5	88.9	89.5	89.3	89.3	89.5	89.9	90.1	89.6	89.9	90.2	89.8	89.8	89.6	89.8	89.8	88.0	89.5	89.9	89.7	89.2	89.3	89.4	89.0	89.0	89.2	89.6	89.2	88.4	99.2	99.2	99.4	99.1	99.3	99.1	99.1	98.9	99.1	100.0	-															
41	COLFH777-15 Finland Aland Islands MZ660166	89.3	88.7	89.3	89.2	89.2	89.3	89.8	89.9	89.5	89.8	90.0	89.6	89.6	89.5	89.6	89.6	88.0	89.3	89.8	89.5	89.0	89.2	89.2	88.9	88.9	89.0	89.5	89.0	88.3	99.4	99.4	99.5	99.2	99.4	99.2	99.2	99.1	99.2	99.8	99.8	-														
42	GCOL3151-16 Germany Thuringia KU919088	89.3	88.7	89.3	89.2	89.2	89.3	89.8	89.9	89.5	89.8	90.0	89.6	89.6	89.5	89.6	89.6	88.0	89.3	89.8	89.5	89.0	89.2	89.2	88.9	88.9	89.0	89.5	89.0	88.3	99.4	99.4	99.5	99.2	99.4	99.2	99.2	99.1	99.2	99.8	99.8	100.0	-													
43	NOCOL068-12 Norway Buskerud	89.3	88.7	89.3	89.2	89.2	89.3	89.8	89.9	89.5	89.8	90.0	89.6	89.6	89.5	89.6	89.6	88.0	89.3	89.8	89.5	89.0	89.2	89.2	88.9	88.9	89.0	89.5	89.0	88.3	99.4	99.4	99.5	99.2	99.4	99.2	99.2	99.1	99.2	99.8	99.8	100.0	100.0	-												
44	NOCLP1630-19 Norway Hedmark	89.3	88.7	89.3	89.1	89.1	89.3	89.8	89.9	89.4	89.8	90.0	89.6	89.6	89.4	89.6	89.6	87.9	89.3	89.8	89.5	89.0	89.1	89.2	88.8	88.8	89.0	89.4	89.0	88.2	99.4	99.4	99.5	99.2	99.4	99.2	99.2	99.1	99.2	99.8	99.8	100.0	100.0	100.0	-											
45	TDAAT1213-20 Austria Tyrol	89.1	88.4	89.1	88.9	88.9	89.1	89.7	89.9	89.4	89.8	90.0	89.5	89.5	89.5	89.5	89.5	87.7	89.2	89.7	89.5	89.0	89.2	89.2	88.7	88.7	88.9	89.4	89.0	88.1	99.4	99.4	99.5	99.2	99.4	99.2	99.2	99.0	99.2	99.8	99.8	100.0	100.0	100.0	100.0	-										
46	GBCL41548-19 Slovakia JX412733	89.2	88.6	89.2	89.0	89.0	89.2	89.6	89.8	89.3	89.6	89.8	89.5	89.5	89.3	89.5	89.5	88.0	89.2	89.6	89.4	88.9	89.0	89.1	88.7	88.7	88.9	89.3	88.8	88.1	99.2	99.2	99.4	99.1	99.3	99.1	99.1	98.9	99.1	99.7	99.7	99.8	99.8	99.8	99.8	99.8	-									
47	LEFIJ2367-14 Finland MZ609611	89.5	88.9	89.5	89.3	89.3	89.5	89.9	90.1	89.6	89.9	90.2	89.8	89.8	89.6	89.8	89.8	88.0	89.5	89.9	89.7	89.2	89.3	89.4	89.0	89.0	89.2	89.6	89.2	88.4	99.5	99.5	99.7	99.4	99.3	99.4	99.4	99.2	99.4	99.7	99.7	99.8	99.8	99.8	99.8	99.8	99.7	-								
48	LEFIJ2369-14 Finland MZ610731	89.2	88.6	89.2	89.0	89.0	89.2	89.6	89.8	89.3	89.6	89.8	89.5	89.5	89.3	89.5	89.5	88.0	89.2	89.6	89.4	88.9	89.0	89.1	88.7	88.7	88.9	89.3	88.8	88.1	99.2	99.2	99.4	99.1	99.3	99.1	99.1	98.9	99.1	99.7	99.7	99.8	99.8	99.8	99.8	99.8	99.7	99.7	-							
49	LEFIJ2374-14 Finland MZ606944	89.2	88.6	89.2	89.0	89.0	89.2	89.6	89.8	89.3	89.6	89.8	89.5	89.5	89.3	89.5	89.5	88.0	89.2	89.6	89.4	88.9	89.0	89.1	88.7	88.7	88.9	89.3	88.8	88.1	99.2	99.2	99.4	99.1	99.3	99.1	99.1	98.9	99.1	99.7	99.7	99.8	99.8	99.8	99.8	99.8	99.7	99.7	100.0	-						
50	NOCOL065-12 Norway Buskerud	89.2	88.6	89.2	89.0	89.0	89.2	89.6	89.8	89.3	89.6	89.8	89.5	89.5	89.3	89.5	89.5	87.7	89.2	89.6	89.4	88.9	89.0	89.1	88.7	88.7	88.9	89.3	88.8	88.1	99.5	99.5	99.7	99.4	99.6	99.4	99.4	99.2	99.4	99.7	99.7	99.8	99.8	99.8	99.8	99.8	99.7	99.7	99.7	99.7	-					
51	NOCOL066-12 Norway Aust-Agder	89.0	88.4	89.0	88.9	88.9	89.3	89.8	89.9	89.5	89.8	90.0	89.6	89.6	89.5	89.6	89.6	88.0	89.3	89.8	89.5	89.0	89.2	89.2	88.9	88.9	89.0	89.5	89.0	88.0	99.1	99.1	99.2	98.9	99.1	98.9	98.9	98.8	98.9	99.5	99.5	99.7	99.7	99.7	99.7	99.7	99.5	99.5	99.5	99.5	99.5	-				
52	PSFOR868-14 France Auvergne-Rhones-Alpes KM286113	89.3	88.7	89.3	89.2	89.2	89.3	89.8	89.9	89.5	89.8	90.0	89.6	89.6	89.5	89.6	89.6	88.0	89.3	89.8	89.5	89.0	89.2	89.2	88.9	88.9	89.0	89.5	89.0	88.3	99.1	99.1	99.2	98.9	99.1	98.9	98.9	98.8	98.9	99.5	99.5	99.7	99.7	99.7	99.7	99.7	99.5	99.5	99.5	99.5	99.5	99.4	-			
53	COLFH1049-15 Finland MZ658831	88.7	88.1	88.7	88.6	88.6	88.7	89.5	89.6	89.2	89.4	89.4	89.3	89.3	89.1	89.0	89.0	87.2	88.7	89.2	88.9	88.4	88.5	88.6	88.3	88.3	88.4	88.9	88.4	87.7	99.4	99.4	99.5	99.2	99.4	99.2	99.2	99.4	99.2	99.2	99.2	99.4	99.4	99.4	99.4	99.4	99.2	99.2	99.2	99.2	99.5	99.1	99.1	-														
54	LEFIJ2373-14 Finland MZ611117	88.7	88.1	88.7	88.6	88.6	88.7	89.5	89.6	89.2	89.4	89.4	89.3	89.3	89.1	89.0	89.0	87.2	88.7	89.2	88.9	88.4	88.5	88.6	88.3	88.3	88.4	88.9	88.4	87.7	99.4	99.4	99.5	99.2	99.4	99.2	99.2	99.4	99.2	99.2	99.2	99.4	99.4	99.4	99.4	99.4	99.2	99.2	99.2	99.2	99.5	99.1	99.1	100.0	-													
55	COLFH297-14 Finland MZ611036	88.9	88.3	88.9	88.7	88.7	88.9	89.6	89.8	89.3	89.6	89.5	89.5	89.5	89.3	89.2	89.2	87.5	88.9	89.3	89.0	88.5	88.7	88.7	88.4	88.4	88.6	88.9	88.6	87.7	99.2	99.2	99.4	99.1	99.3	99.1	99.1	99.2	99.1	99.1	99.1	99.2	99.2	99.2	99.2	99.2	99.1	99.1	99.1	99.1	99.4	98.9	98.9	99.8	99.8	-												
56	COLFH1048-15 Finland MZ659482	89.3	88.6	88.9	89.0	89.0	89.2	89.6	89.8	89.3	89.4	89.8	89.5	89.5	89.3	89.5	89.5	88.0	89.2	89.6	89.0	88.7	88.8	88.9	88.7	88.7	88.9	89.3	88.6	88.0	99.2	99.2	99.1	99.4	99.3	99.1	99.1	98.9	99.1	98.8	98.8	98.9	98.9	98.9	98.9	98.9	98.8	98.8	98.8	98.8	99.1	98.6	98.9	98.9	98.9	98.8	-											
57	FBCOB988-10 Germany Rhineland-Palatinate HQ954434	89.2	88.4	88.7	88.9	88.9	89.0	89.5	89.6	89.2	89.4	89.7	89.3	89.3	89.1	89.3	89.3	87.7	89.0	89.5	88.9	88.7	88.8	88.9	88.6	88.6	88.7	89.2	88.6	87.8	99.1	99.1	98.9	99.2	99.1	98.9	98.9	98.8	98.9	98.6	98.6	98.8	98.8	98.8	98.8	98.7	98.6	98.6	98.6	98.6	98.9	98.5	98.5	98.8	98.8	98.6	99.5	-										
58	COLFC192-12 Finland Lapland KJ965040	89.0	88.6	89.2	89.0	89.0	88.7	89.3	89.5	89.0	88.6	89.0	89.5	89.5	89.1	89.2	89.2	87.5	89.0	89.5	88.6	88.5	88.3	88.6	88.6	88.6	88.7	88.3	87.7	87.8	93.9	93.9	94.1	93.8	93.6	93.8	93.7	93.9	93.7	93.8	93.8	93.6	93.6	93.6	93.6	93.5	93.4	93.8	93.8	93.8	93.8	93.6	93.6	93.9	93.9	93.8	93.8	93.3	-									
59	COLFE069-12 Finland KJ964307	89.0	88.6	89.2	89.0	89.0	88.7	89.3	89.5	89.0	88.6	89.0	89.5	89.5	89.1	89.2	89.2	87.5	89.0	89.5	88.6	88.5	88.3	88.6	88.6	88.6	88.7	88.3	87.7	87.8	93.9	93.9	94.1	93.8	93.6	93.8	93.7	93.9	93.7	93.8	93.8	93.6	93.6	93.6	93.6	93.5	93.4	93.8	93.8	93.8	93.8	93.6	93.6	93.9	93.9	93.8	93.8	93.3	100.0	-								
60	COLFH776-15 Finland MZ657676	88.9	88.4	89.0	88.9	88.9	88.6	89.2	89.3	88.9	88.6	88.9	89.3	89.3	89.1	89.0	89.0	87.2	88.9	89.3	88.4	88.5	88.3	88.6	88.4	88.4	88.6	88.1	87.7	87.7	93.8	93.8	93.9	93.6	93.5	93.6	93.5	93.8	93.6	93.6	93.6	93.4	93.4	93.4	93.4	93.3	93.3	93.6	93.6	93.6	93.6	93.4	93.4	93.8	93.8	93.6	93.6	93.1	99.8	99.8	-							
61	LEFIJ2371-14 Finland MZ607436	88.9	88.4	89.0	88.9	88.9	88.6	89.2	89.3	88.9	88.6	88.9	89.3	89.3	89.1	89.0	89.0	87.2	88.9	89.3	88.4	88.5	88.3	88.6	88.4	88.4	88.6	88.1	87.7	87.7	93.8	93.8	93.9	93.6	93.5	93.6	93.5	93.8	93.6	93.6	93.6	93.4	93.4	93.4	93.4	93.3	93.3	93.6	93.6	93.6	93.6	93.4	93.4	93.8	93.8	93.6	93.6	93.1	99.8	99.8	100.0	-						
62	COLFH1324-16 Finland MZ659395	88.7	88.3	88.9	88.7	88.7	88.4	89.0	89.2	88.7	88.4	88.7	89.2	89.2	89.0	88.9	88.9	87.0	88.7	89.2	88.3	88.7	88.5	88.7	88.6	88.6	88.7	88.0	87.5	87.5	93.6	93.6	93.8	93.4	93.3	93.4	93.4	93.6	93.4	93.4	93.4	93.3	93.3	93.3	93.3	93.2	93.1	93.4	93.4	93.4	93.4	93.3	93.3	93.6	93.6	93.4	93.4	93.0	99.7	99.7	99.8	99.8	-					
63	LEFIJ2368-14 Finland MZ609720	89.0	88.6	89.2	89.0	89.0	88.7	89.3	89.5	89.0	88.8	89.0	89.5	89.5	89.3	89.2	89.2	87.2	89.0	89.5	88.6	88.7	88.5	88.7	88.6	88.6	88.7	88.3	87.7	87.8	93.8	93.8	93.9	93.6	93.5	93.6	93.5	93.4	93.6	93.6	93.6	93.4	93.4	93.4	93.4	93.3	93.3	93.6	93.6	93.6	93.6	93.4	93.4	93.4	93.4	93.3	93.6	93.1	99.5	99.5	99.7	99.7	99.5	-				
64	COLFH1327-16 Finland MZ659144	89.2	89.0	89.3	89.2	89.2	89.2	89.2	89.3	88.9	88.4	88.9	89.3	89.3	89.0	89.3	89.3	87.5	89.2	89.6	88.7	88.5	88.3	88.2	88.6	88.6	88.7	88.9	87.9	88.0	93.6	93.6	93.8	93.4	93.3	93.4	93.4	93.6	93.4	93.8	93.8	93.6	93.6	93.6	93.6	93.5	93.4	93.8	93.8	93.8	93.4	93.6	93.6	93.6	93.6	93.4	93.4	93.0	98.3	98.3	98.2	98.2	98.0	97.9	-			
65	LEFIJ2372-14 Finland MZ609121	89.0	88.9	89.2	89.0	89.0	89.0	89.3	89.2	88.7	88.2	88.7	89.2	89.2	88.8	89.2	89.2	87.2	89.0	89.5	88.6	88.4	88.2	88.1	88.7	88.7	88.9	88.7	87.7	87.8	93.4	93.4	93.6	93.3	93.2	93.3	93.2	93.4	93.3	93.6	93.6	93.4	93.4	93.4	93.4	93.3	93.3	93.6	93.6	93.6	93.3	93.4	93.4	93.4	93.4	93.3	93.3	92.8	98.2	98.2	98.0	98.0	97.9	97.7	99.8	-		
66	COLFH773-15 Finland MZ660688	89.0	88.9	89.2	89.0	89.0	89.3	89.3	89.5	89.0	88.6	89.0	89.5	89.5	89.1	89.5	89.5	87.7	89.3	89.8	88.9	88.7	88.5	88.4	88.7	88.7	88.9	88.9	88.1	87.8	93.8	93.8	93.9	93.6	93.5	93.6	93.5	93.8	93.6	93.9	93.9	93.8	93.8	93.8	93.7	93.7	93.6	93.9	93.9	93.9	93.6	93.8	93.8	93.8	93.8	93.6	93.6	93.1	98.2	98.2	98.0	98.0	97.9	97.7	99.7	99.5	-	
67	COLFH774-15 Finland MZ656536	89.0	88.9	89.2	89.0	89.0	89.3	89.3	89.5	89.0	88.6	89.0	89.5	89.5	89.1	89.5	89.5	87.7	89.3	89.8	88.9	88.7	88.5	88.4	88.7	88.7	88.9	88.9	88.1	87.8	93.8	93.8	93.9	93.6	93.5	93.6	93.5	93.8	93.6	93.9	93.9	93.8	93.8	93.8	93.7	93.7	93.6	93.9	93.9	93.9	93.6	93.8	93.8	93.8	93.8	93.6	93.6	93.1	98.2	98.2	98.0	98.0	97.9	97.7	99.7	99.5	100.0	-
